# Associations of Domestic Violence Types with Self-Harm in Women Using Mental Health Services in South-East London

**DOI:** 10.1192/bjo.2026.11148

**Published:** 2026-06-30

**Authors:** Rojbin Yigit, Roxanne Keynejad, Vishal Bhavsar

**Affiliations:** King’s College London, London, United Kingdom

## Abstract

**Aims:**

Estimate associations of domestic violence (DV; physical, sexual, emotional, financial) with Emergency Department (ED) attendance for self-harm in women using mental health services.

**Methods:**

We analysed data from the Clinical Record Interactive Search (CRIS),an electronic health record database (EHR) from South London, of women who used South London and Maudsley (SLaM) NHS Trust mental health services. We used a previously developed and validated natural language processing (NLP) algorithm to ascertain identification of DV types occurring within the first year of care and at any time. ED attendances for self-harm were gathered from hospital episodes statistics (HES) data linked tothe EHR database. We adjusted for variables relating to DV and self-harm such as age, ethnicity and psychiatric diagnosis to estimate associations of DV types with ED attendances for self-harm.

**Results:**

Between 2007 and 2020, 240,180 women used services. Missing data was excluded, data from 79,025 women were analysed. Overall, 2670 (3.4%) of women had ED self-harm attendance. The most common DV types recorded at any time were physical (28.8%) and emotional (13.7%). Sexual DV had the strongest association with self-harm. After adjusting for factors that can affect self-harm and DV, the strongest association was for physical DV. The strongest association in the first 12 months after referral was for financial DV.

**Conclusion:**

Women with a history of DV are more likely to use mental health services. Associations between DV types with self-harm are unexplored. Women accessing services who experience DV are more likely to attend hospital due to self-harm than those not experiencing DV. Physical and emotional DV were the most common types. There were associations between self-harm and most DV types. These findings can inform interventions and service improvements.

